# Predictors of sexual attitudes in arranged marriages: the mediating role of sexual intercourse frequency

**DOI:** 10.1093/sexmed/qfag085

**Published:** 2026-09-30

**Authors:** Aysu Yildiz Karaahmet

**Affiliations:** Department of Midwifery, Faculty of Health Sciences, Biruni University, Zeytinburnu, Istanbul 34015, Türkiye

**Keywords:** arranged marriage, sexual attitudes, sexual intercourse frequency, mediation analysis, Structural Equation Modeling

## Abstract

**Introduction:**

Sexual attitudes in arranged marriages are shaped by sociocultural norms, gender roles, and intramarital relational dynamics, yet the mechanisms underlying these attitudes remain insufficiently understood. This study aimed to examine the associations between sociodemographic and marital characteristics and sexual attitudes among individuals in arranged marriages, and to explore the mediating role of sexual intercourse frequency within this population.

**Methods:**

This cross-sectional study included 520 married adults (280 women and 240 men) who had entered arranged marriages. Data were collected through an online survey using a sociodemographic information form and the Hendrick Sexual Attitudes Scale (HSAS). Group comparisons were performed using independent samples *t*-tests and one-way analysis of variance (ANOVA), and Structural Equation Modeling (SEM) was used to examine direct and indirect associations among variables. The primary outcome was sexual attitude score measured by the HSAS.

**Results:**

Male gender, younger age, and higher educational level were significantly associated with more positive sexual attitudes (*P* <.05). The structural model demonstrated good fit indices (χ^2^/df = 2.45, CFI = 0.948, TLI = 0.939, RMSEA = 0.051, SRMR = 0.044) and explained 38% of the variance in sexual attitudes. Structural Equation Modeling showed that male gender (β = 0.16) and higher education (β = 0.21) were significantly associated with more positive sexual attitudes, whereas older age (β = −0.12) was significantly associated with less positive sexual attitudes. Interventions aimed at supporting regular sexual interaction and addressing relational changes associated with long-term marriage and parenthood may help maintain positive sexual attitudes in arranged marriages.

**Conclusion:**

The findings suggest that sexual attitudes among individuals in arranged marriages are associated with both sociodemographic characteristics and intramarital sexual interaction patterns. Within the proposed model, sexual intercourse frequency emerged as a significant mediator of these associations.

## Introductıon

Sexuality is a multifaceted and dynamic phenomenon encompassing the biological, psychological, and social dimensions of human life. Sexual attitudes, defined as the totality of individuals' beliefs, values, and emotional responses to sexuality, are shaped by cultural norms, gender roles, and individual experiences.^1^ In the literature, sexual attitudes are considered one of the strongest psychological constructs predicting individuals' sexual behavior and are regarded as a cornerstone of marital harmony.^2^^,^^3^ In societies like Turkey, where collectivist and individualistic values coexist, the ways in which individuals enter the institution of marriage play a critical role in the development of these attitudes.

Arranged marriage is a traditional system where family approval and mediation mechanisms are central to spouse selection. Despite modernization and technological advancements, this form of marriage maintains its cultural continuity not only in Turkey but also in a wide geographical area such as South Asia and the Middle East.^4^^,^^5^ While sexual interaction in love marriages usually begins during the pre-marital courtship period, in arranged marriages, the exploration of sexuality generally intensifies in the post-marriage period. This situation differentiates the pathways individuals take to establish sexual intimacy.^6^^,^^7^

The most significant differentiation in sexual attitudes is gender-based. Sexual Strategies Theory^8^ and Social Role Theory^9^ emphasize that men evaluate sexuality from a more physical (instrumental) perspective, while women integrate sexuality with emotional commitment and relationship quality.^10^^,^^11^ Furthermore, it has been found that education helps individuals develop more liberal attitudes by increasing their level of sexual knowledge^12^^,^^13^ while age has a restrictive effect on attitudes due to conservatism and the cohort effect.^14^

When marital dynamics are considered, the Sexual Habituation Hypothesis, characterized by the decrease in the “honeymoon effect,” argues that sexual desire tends to decrease as the duration of marriage increases.^15^ The most important life event that accelerates this decrease is the transition to parenthood. The study by Doss *et al.* shows that having children reduces romantic interaction between spouses.^16^ In this process, the frequency of sexual intercourse is a source that refreshes an individual's perspective on sexuality, according to the Self-Perception Theory.^17^ However, the indirect effects of these factors in arranged marriages, particularly the mediating role of sexual intercourse frequency, have not yet been empirically modeled.

This study aimed to examine the associations between sociodemographic and marital characteristics and sexual attitudes among individuals who had entered arranged marriages using a Structural Equation Modeling (SEM) framework. The study was designed to identify patterns of association within a sample of individuals in arranged marriages rather than to compare arranged marriages with other forms of marriage. Specifically, the study sought to address the following testable research questions: Are gender, age, and education level significantly associated with sexual attitudes among individuals in arranged marriages? Does the frequency of sexual intercourse mediate the associations of marital duration and parenthood with sexual attitudes among individuals in arranged marriages? By addressing these questions, the study aimed to contribute to the literature by examining sexual dynamics within a traditional marriage context using SEM.

## Methods

### Study design and setting

This study employed a cross-sectional, observational design to examine sexual attitudes among individuals who had entered marriage through arranged processes. Data were collected via an online survey administered between July 1 and October 1, 2025. A cross-sectional approach was deemed appropriate given the exploratory nature of the research questions and the aim of identifying sociodemographic and marital correlates of sexual attitudes within arranged marriages. The study was reported in accordance with the Strengthening the Reporting of Observational Studies in Epidemiology (STROBE) statement for cross-sectional studies, and the completed STROBE checklist is provided as a supplementary file.

### Participants and eligibility criteria

The study sample consisted of 520 married adults (280 women and 240 men) who self-reported having entered their current marriage through an arranged process. Eligibility criteria included:

(1) being 18 years of age or older,
(2) being currently married,
(3) having entered marriage through an arranged mechanism, and
(4) the ability to read and understand Turkish.

Participants who did not provide informed consent or who reported marriage through a fully self-initiated romantic relationship were excluded.

For the purposes of this study, arranged marriage was operationally defined as a marriage in which partner selection or the initiation of the marital relationship occurred through an arranged process rather than exclusively through a self-initiated premarital romantic relationship. The questionnaire distinguished arranged marriages from self-initiated romantic marriages and also included an option for marriages initiated through online matchmaking platforms specifically intended to facilitate partner matching for marriage. This category referred specifically to marriage-oriented matchmaking services rather than general online dating or social networking platforms. However, no participants in the present sample reported having entered their marriage through this type of online matchmaking platform. Therefore, the analyzed sample consisted exclusively of participants who self-identified their marriage as arranged.

### Sampling procedure and data collection

Participants were recruited using a snowball sampling strategy, which was considered appropriate given the cultural sensitivity of sexuality-related topics and the challenges associated with accessing individuals in arranged marriages through probability-based sampling methods. Initial participants were contacted through the researchers’ professional and social networks and were invited to share the survey link with other eligible individuals.

Data were collected using a secure online survey platform. Prior to participation, respondents were provided with detailed information regarding the study aims, voluntary nature of participation, confidentiality measures, and their right to withdraw at any time without consequence. Electronic informed consent was obtained before access to the questionnaire. The average completion time was approximately 10-12 minutes, and no financial or material incentives were offered.

### Measures

#### Sociodemographic and marital characteristics

Sociodemographic and marital data were collected using a structured questionnaire developed by the researchers. Variables included age, gender, educational attainment, employment status, perceived socioeconomic status, place of residence (urban or rural), duration of marriage (in years), parental status (having children: yes/no), and marriage initiation was assessed as arranged, self-initiated romantic, or through an online matchmaking platform specifically intended to facilitate partner matching for marriage.

#### Sexual attitudes

Sexual attitudes were assessed using the Hendrick Sexual Attitudes Scale (HSAS).^1^^,^^18^ The HSAS is a widely used self-report instrument designed to assess individuals’ cognitive and affective orientations toward sexuality. The short-form version used in this study consists of 23 items and retains a 4-factor structure encompassing: (1) permissiveness (attitudes toward casual sexuality), (2) birth control (responsible and tolerant sexuality), (3) communion (idealistic and relational sexuality), and (4) instrumentality (biological and utilitarian views of sexuality).^1^^,^^18^

Items are rated on a Likert-type scale, with higher scores indicating more permissive or positive sexual attitudes. In line with prior conceptualizations, permissiveness and instrumentality primarily reflect self-oriented sexual attitudes, whereas communion and birth control reflect partner-oriented and relational perspectives.^1^

In the present study, internal consistency reliability was assessed using Cronbach’s alpha coefficients for the total scale and each subdimension, all of which demonstrated acceptable reliability. The complete questionnaire administered in this study, including the researcher-developed sociodemographic and marital information form and the HSAS, is provided as Supplementary File S1.

### Statistical analysis

Statistical analyses were performed using IBM SPSS Amos, version 24.0 (IBM Corp., Armonk, NY, United States)^19^ and IBM SPSS Statistics for Windows, version 26.0 (IBM Corp., Armonk, NY, United States).^20^ Before the main analyses, data were examined for missing values, outliers, and normality assumptions. Descriptive statistics, including mean, standard deviation, and frequencies, were calculated to summarize sample characteristics. Group differences in sexual attitude scores were examined using an independent samples *t-*test and Tukey post-hoc comparative one-way ANOVA. Although employment status, perceived socioeconomic status, and place of residence were collected as descriptive variables, they were not retained in the final structural model because preliminary analyses did not demonstrate significant associations with sexual attitudes. Therefore, the SEM focused on variables that were theoretically relevant and empirically supported in the preliminary analyses. Because the study was designed to examine associations within a sample of individuals in arranged marriages, no comparison analyses with other marital types were performed. The data analysis was based on a 2-stage SEM approach. First, Confirmatory Factor Analysis (CFA) was performed to assess the validity and reliability of the measurement model. Construct validity was evaluated using Compound Reliability (CR) and Mean Explained Variance (AVE) values. Second, the structural model was tested using the Maximum Likelihood (ML) estimation method to examine direct and indirect pathways between sociodemographic factors, marital variables, and sexual attitudes. Model fit was assessed based on multiple indices: a chi-square ratio of degrees of freedom less than 3.0, Comparative Fit Index (CFI) and Tucker-Lewis Index (TLI) values ​​greater than 0.90, and a Root Mean Square Error of Approximation (RMSEA) value less than 0.08. To test the mediating role of frequency of sexual intercourse, a bootstrapping procedure with 5000 samples was used to calculate 95% CIs for indirect effects. If the 95% CI did not include zero, the indirect effect was considered statistically significant. For all other tests, the statistical significance level was set at *P* <.05.

### Ethical considerations

The study was conducted in accordance with the principles of the Declaration of Helsinki. Ethical approval was obtained from the Biruni University Institutional Ethics Committee (Approval No: 2024-BİAEK-07-10; Date: 24 February 2025). Participation was entirely voluntary, and electronic informed consent was obtained from all participants prior to data collection. All data were collected anonymously and stored securely, with access restricted to the research team.

## Results

The research sample consisted of 520 participants with a balanced distribution in terms of gender and education level (Table 1). The analyses showed that men (3.42 ± 0.44) had significantly higher sexual attitude scores than women (3.30 ± 0.41) (*P* = .006). Significant differences were also found according to age, education, and marital characteristics. Younger participants, those with higher education levels, and those without children were found to have more positive sexual attitudes (*P* < .05). In addition, a higher frequency of sexual intercourse was significantly associated with more positive/permissive sexual attitudes (*P* < .001). Employment status, perceived socioeconomic status, and place of residence were also examined in the preliminary analyses; however, no statistically significant associations with sexual attitudes were observed (all *P* > .05). Therefore, these variables were not retained in the final structural equation model.

**Table 1:** Sociodemographic and marital characteristics and their relationship with sexual Attitudes (*N* = 520).

Variable	Category	*n* (%)	Mean ± SD	Test	*P*
Gender	Female	280 (53.8)	3.30 ± 0.41	t	.006
	Male	240 (46.2)	3.42 ± 0.44		
Age (years)	18-29	142 (27.3)	3.45 ± 0.41	F	.018
	30-39	228 (43.8)	3.38 ± 0.43		
	≥40	150 (28.9)	3.26 ± 0.42		
Education level	≤High school	260 (50.0)	3.21 ± 0.43	t	.001
	University or higher	260 (50.0)	3.46 ± 0.42		
Duration of marriage	<5 years	214 (41.2)	3.48 ± 0.40	F	.002
	5-10 years	168 (32.3)	3.34 ± 0.41		
	>10 years	138 (26.5)	3.18 ± 0.44		
Children	Yes	304 (58.5)	3.27 ± 0.43	t	.001
	No	216 (41.5)	3.48 ± 0.40		
Freq. of intercourse	≥1 per week	218 (41.9)	3.55 ± 0.39	F	.001
	1-3 per month	192 (36.9)	3.29 ± 0.41		
	<1 per month	110 (21.2)	3.10 ± 0.45		

Abbreviation: SD, standart sapma.

Statistically significant effects are indicated by the corresponding *P* values (*P* < .05).

The validity and reliability of the Hendrick Sexual Attitude Scale were evaluated with CFA. As seen in Table 2, all dimensions of Permissiveness, Birth Control, Cohabitation, and Instrumentalism showed high internal consistency (Cronbach's alpha > .80). Composite Reliability (CR) values ​​above .70 and AVE values ​​above .50 have proven the structural validity of the scale.

**Table 2:** Measurement model results: validity and reliability (CFA).

Constructs	Cronbach’s α	CR	AVE	1	2	3	4
1. Permissiveness	0.84	0.85	0.58	(0.76)				
2. Birth control	0.82	0.83	0.56	0.34	(0.74)			
3. Communion	0.86	0.87	0.62	0.42	0.48	(0.78)		
4. Instrumentality	0.81	0.82	0.53	0.51	0.29	0.36	(0.72)	

Abbreviations: α, Cronbach's alpha; AVE, Average Variance Extracted; CR, Composite Reliability.

Values on the diagonal (in parentheses) represent the square root of the AVE for each construct. Off-diagonal values represent Pearson correlation coefficients among the latent constructs.

Structural relationships between variables were examined using path analysis (Table 3). The model visualized in Figure 1 revealed that male gender (β = 0.16) and higher education (β = 0.21) were positively associated with sexual attitudes (*P* < .001). In contrast, older age was negatively associated with sexual attitudes (β = -0.12, *P* = .004).

**Table 3:** Structural path coefficients: direct and indirect effects.

Path	Stand. β	SE	*t*-value	*P*
Direct effects				
Age → sexual attitude	-0.12	0.04	-2.85	.004
Gender (male) → sexual attitude	0.16	0.03	4.12	<.001
Education → sexual attitude	0.21	0.03	5.20	<.001
Intercourse freq. → sexual attitude	0.28	0.04	6.55	<.001
Children (yes) → Intercourse freq.	-0.25	0.05	-5.12	<.001
Indirect (mediation) effects				
Age → intercourse freq. → sexual att.	-0.042	0.01	-2.15	.031
Children → intercourse freq. → sexual att.	-0.070	0.02	-3.88	<.001

Abbreviations: β, standardized regression coefficient; SE, standard error. Statistically significant effects are indicated by the corresponding *P* values (*P* < .05, *P* < .01, *P* < .001).

**Figure 1:**
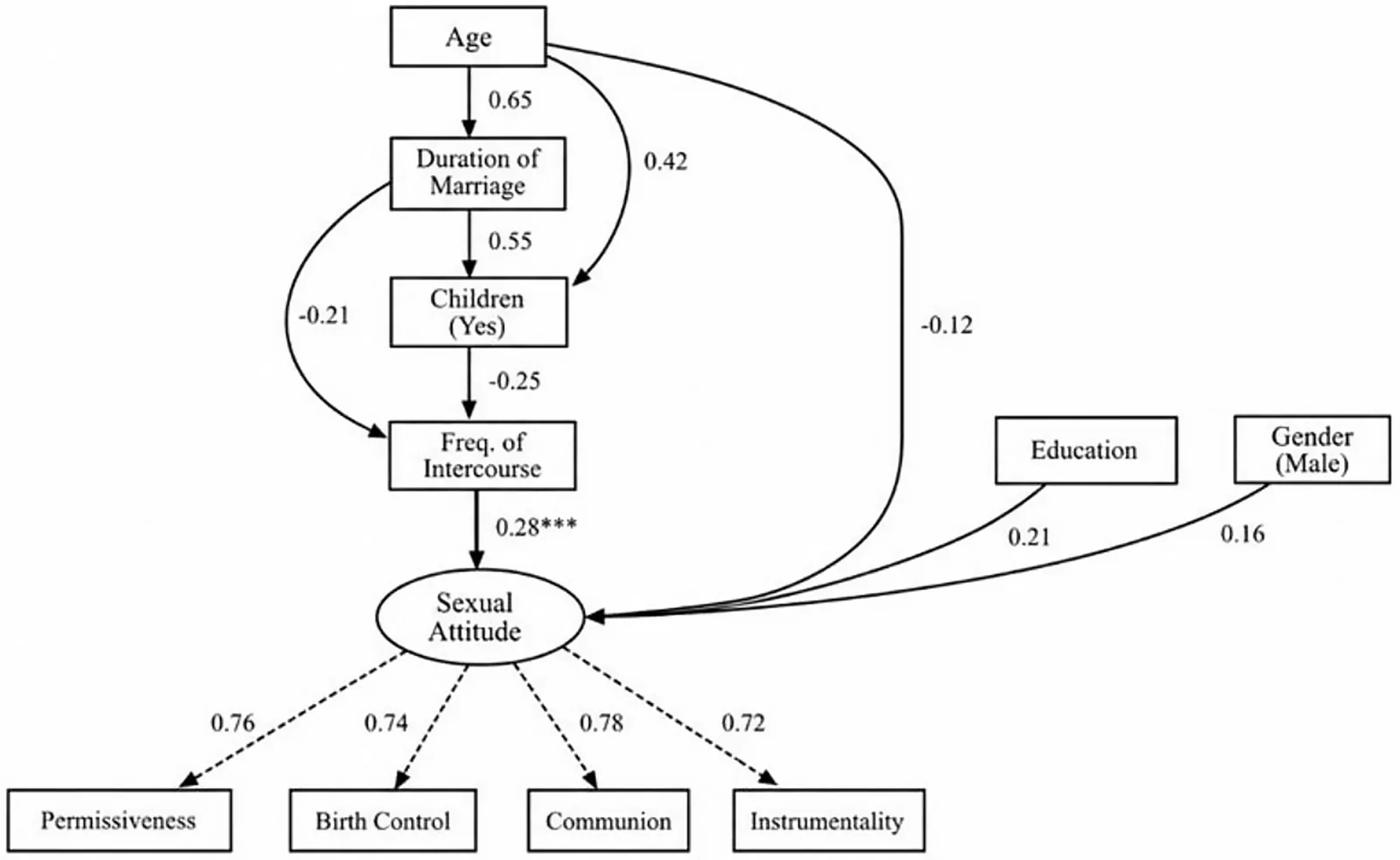
Path diagram of the structural equation model illustrating the direct and indirect effects of sociodemographic factors and marital characteristics on the sub-dimensions of sexual attitudes. Values on the paths represent standardized regression coefficients (B). Solid lines indicate structural paths, whereas dashed lines indicate the standardized loadings of the sexual attitude construct on its sub-dimensions. ^***^P < .001.

According to the mediation analysis results, which are the focus of the model, longer marriage duration (β = -0.21) and having children (β = -0.25) were significantly associated with lower frequency of sexual intercourse. On the other hand, a higher frequency of sexual intercourse was significantly associated with more positive sexual attitudes (β = 0.28). The mediation analysis indicated that the associations of longer marital duration and parenthood with sexual attitudes were statistically mediated by the frequency of sexual intercourse.

Finally, the statistical performance of the structural model was evaluated. As summarized in Table 4, the model showed good fit with the data (Chi-square/df = 2.45, CFI = .948, TLI = .939, RMSEA = .051, SRMR = .044). In addition, the model accounted for 38% of the variance in sexual attitudes.

**Table 4:** Model fit indices and explained variance.

Fit index	Model value	Threshold (cut-off)	Decision
Chi-square/df	2.45	<3.0	Good fit
CFI	.948	>.90	Acceptable
TLI	.939	>.90	Acceptable
RMSEA	.051	<.08	Good fit
SRMR	.044	<.08	Good fit
R-squared (sexual attitude)	38%	-	-

Abbreviations: CFI, Comparative Fit Index; SRMR, Standardized Root Mean Square Residual; TLI, Tucker–Lewis Index; RMSEA, Root Mean Square Error of Approximation.

## Discussion

This study investigated the predictors of sexual attitudes and the mediating role of the frequency of sexual intercourse among individuals who entered arranged marriages using SEM. The findings reveal that sexual attitudes are shaped not only by relatively stable sociodemographic characteristics but also by dynamic intramarital processes, particularly patterns of sexual interaction between spouses. The results therefore highlight that sexual attitudes in marriage are not static constructs but evolve through ongoing relational experiences. Because the study included only individuals in arranged marriages, the findings should be interpreted within this marital context and should not be considered evidence of differences between arranged and love marriages.

The first key finding of the study is that men reported significantly higher permissive and instrumental sexual attitude scores than women (Table 1). This finding is consistent with previous literature explained within the framework of Sexual Strategies Theory^8^ and Social Role Theory.^9^ According to Sexual Strategies Theory, men are more likely to approach sexuality from a physical or instrumental perspective, whereas women tend to associate sexuality with emotional intimacy and relational commitment.^8^ Similarly, Social Role Theory suggests that culturally shaped gender norms influence how men and women interpret and express sexuality.^9^ The meta-analysis conducted by Petersen and Hyde also demonstrates that gender schemas influence men to approach sexuality more autonomously and instrumentally, whereas women often integrate sexuality with emotional closeness and relational harmony.^10^ These gender-based differences may become more visible in traditional marital contexts such as arranged marriages.^21^

In Turkey, where collectivist family structures and traditional marital norms continue to shape relationship dynamics, arranged marriages remain an important social institution in some segments of society.^4^^,^^5^ Within such cultural contexts, expectations related to gender roles, modesty norms, and family involvement in marital decisions may further reinforce differences in sexual attitudes between men and women. Therefore, the findings of this study should also be interpreted within the broader sociocultural environment in which sexuality is negotiated.

Another important finding is the strong positive effect of education level on sexual attitudes. Individuals with higher education levels tend to adopt more liberal, rational, and knowledge-based perspectives toward sexuality. Education may increase access to sexual health information, promote critical thinking about traditional norms, and reduce taboos surrounding sexuality. These findings are consistent with previous research suggesting that higher educational attainment is associated with more open and positive sexual attitudes.^13^ In contrast, the negative association between age and sexual attitudes observed in the present study may reflect both biological changes and generational differences in sexual values. As suggested in previous studies, older cohorts may have been socialized within more conservative sexual norms compared with younger generations.^14^

One of the most important contributions of this study is the demonstration that the effects of marital duration and parenthood on sexual attitudes occur indirectly through the frequency of sexual intercourse (Figure 1, Table 3). The results show that longer marital duration and having children significantly reduce the frequency of sexual intercourse, which in turn negatively affects sexual attitudes. This finding supports the concept of the sexual habituation effect, which suggests that sexual novelty and desire may gradually decrease in long-term relationships.^15^ However, the present findings indicate that this decline should not be interpreted solely as a reduction in behavior but also as a process that reshapes individuals’ attitudes toward sexuality.

The transition to parenthood represents a major life transition that can alter couple dynamics. As highlighted in previous research, becoming parents often shifts couples’ focus from their spousal relationship toward parenting responsibilities, reducing the time and emotional energy available for intimate interaction.^16^^,^^22^ In the present study, the frequency of sexual intercourse emerged as a strong predictor of sexual attitudes, explaining a substantial portion of the variance in the model. This finding can be interpreted through the lens of Self-Perception Theory, which proposes that individuals infer their attitudes partly from their own behaviors.^17^ When couples maintain regular sexual interaction, sexuality may continue to be perceived as a meaningful and positive component of their relationship.^3^

Overall, the model explained 38% of the variance in sexual attitudes, suggesting that both sociodemographic factors and relational dynamics play a significant role in shaping sexual perspectives within arranged marriages. These findings provide important insights into sexual attitudes within this marital context and highlight the importance of considering behavioral interaction patterns alongside demographic variables. However, because no comparison group of individuals in love marriages was included, the present findings should be interpreted as describing associations observed within arranged marriages rather than as evidence that these associations are unique to arranged marriages. Future comparative studies including different marital contexts are needed to determine whether these patterns differ according to the type of marriage.

### Limitations

Despite its contributions, this study has several limitations that should be acknowledged. First, the cross-sectional design limits the ability to establish causal relationships between the variables. Although the structural model suggests directional associations, longitudinal studies would be required to determine causal pathways more clearly.

Second, the study did not include a comparison group of individuals who entered marriage through autonomous romantic relationships (love marriages). Therefore, the findings should not be interpreted as demonstrating differences between arranged and love marriages, but rather as describing associations observed within an arranged-marriage sample. Future comparative studies are needed to determine whether these associations differ across marital contexts.

Third, the use of snowball sampling may limit the generalizability of the findings. Participants were recruited through social networks, which may have resulted in a sample that does not fully represent all individuals in arranged marriages.

Fourth, different forms of arranged marriage were analyzed as a single operational category. Because the online-assisted subgroup was small, separate subgroup analyses were not feasible. Future studies with larger samples should investigate whether sexual attitudes differ across specific forms of arranged marriage.

Fifth, the study relied on self-reported measures. Because sexuality is a sensitive topic, participants’ responses may have been influenced by social desirability bias or reluctance to disclose personal experiences fully.

Future studies could address these limitations by employing longitudinal designs, more diverse sampling strategies, and mixed-method approaches. Including additional variables such as sexual satisfaction, marital satisfaction, and communication patterns may also provide a more comprehensive understanding of sexual dynamics in arranged marriages.

## Conclusion

This study suggests that sexual attitudes among individuals in arranged marriages are associated with both individual sociodemographic characteristics and relational processes within marriage. In particular, the mediating role of sexual intercourse frequency indicates that the gradual decline in sexual attitudes over time may largely be associated with reductions in sexual interaction between spouses.

These findings highlight that maintaining sexual interaction is not only related to physical intimacy but also plays an important role in shaping individuals’ perceptions and attitudes toward sexuality within marriage. From a practical perspective, the results suggest that marital counseling and sexual health interventions in traditional marital contexts should address both attitudinal and behavioral aspects of sexuality. Supporting couples in maintaining healthy patterns of sexual interaction may contribute to sustaining positive sexual attitudes and overall marital well-being. These findings should be interpreted within the context of arranged marriages and do not permit direct comparisons with other forms of marriage.

## Supplementary Material

Supplementary_material_qfag085

## Data Availability

All data generated or analyzed during this study are included in this published article and its supplementary materials.

## References

[1] Hendrick C, Hendrick SS, Reich DA. The brief sexual attitudes scale. Journal of Sex Research. 2006;43(1):76–86. 10.1080/00224490609552300 16817070

[2] Sprecher S . The relation between social exchange variables and sexual choices, influences, and satisfaction. Journal of Sex Research. 1989;26(1):1–21.

[3] Muise A, Schimmack U, Impett EA. Sexual frequency predicts greater well-being, but more is not always better. Soc Psychol Personal Sci. 2016;7(1):295–302. 10.1177/1948550615616462

[4] Lalonde RN, Hynie M, Pannu M, Tatla S. The role of culture in interpersonal relationships: do second generation South Asian Canadians want a traditional partner? J Cross-Cult Psychol. 2004;35(5):503–524. 10.1177/0022022104268386

[5] Allendorf K, Ghimire DJ. Determinants of marital quality in an arranged marriage society. J Marriage Fam. 2013;75(5):1181–1199. 10.1016/j.ssresearch.2012.09.002 PMC3711098 23146598

[6] Xiaohe X, Whyte MK. Love matches and arranged marriages: a Chinese replication. J Marriage Fam. 1990;52(3):709–722. 10.2307/352936

[7] Epiphaniou E, Kalkan M, Kaya M. Marital satisfaction and sexual dynamics in arranged marriages: a qualitative and quantitative synthesis. J Fam Issues. 2021;42(5):1018–1042.

[8] Buss DM, Schmitt DP. Sexual strategies theory: an evolutionary perspective on human mating. Psychol Rev. 1993;100(2):204–232. 10.1037/0033-295X.100.2.204 8483982

[9] Eagly AH, Wood W. The origins of sex differences in human behavior: evolved dispositions versus social roles. Am Psychol. 1999;54(6):408–423. 10.1037/0003-066X.54.6.408

[10] Petersen JL, Hyde JS. A meta-analytic review of research on gender differences in sexuality, 1993–2007. Psychol Bull. 2010;136(1):21–38. 10.1037/a0017504 20063924

[11] Conley TD, Ziegler A, Moors AC. Backlash from the bedroom: stigma and women's sexual orientations. J Pers Soc Psychol. 2011;100(2):309–321. 10.1037/a0022152 21171789

[12] Wiederman MW . Extramarital sex: prevalence and correlates in a national sample. Journal of Sex Research. 1997;34(2):167–174. 10.1080/00224499709551881

[13] Kontula O . Between sexual desire and reality: the evolution of European sexual lifestyles. Journal of Sex Research. 2009;46(2-3):133–144.

[14] Twenge JM, Sherman RA, Wells BE. Sexual inactivity during young adulthood is more common among US millennials and iGen. Arch Sex Behav. 2017;46:433–440. 10.1007/s10508-016-0798-z 27480753

[15] Dewitte M, Mayer A, Lowenstein L, Janssen E. Sexual satisfaction and relationship quality in long-term relationships: the role of sexual habituation. Journal of Sex Research. 2020;57(1):1–13.

[16] Doss BD, Rhoades GK, Stanley SM, Markman HJ. The effect of the transition to parenthood on relationship quality: an 8-year prospective study. J Pers Soc Psychol. 2009;96(3):601–619. 10.1037/a0013969 PMC2702669 19254107

[17] Bem DJ . Self-perception theory. In: Berkowitz L ed. Advances in Experimental Social Psychology. Vol 6. Academic Press; 1972: 1–62.

[18] Gülüm İV, Dağ İ. Hendrick Cinsel Tutumlar Ölçeği Kısa Formunun Türk örneklemindeki psikometrik özellikleri. Anadolu Psikiyatri Dergisi. 2012;13(2):154–162.

[19] Arbuckle JL . IBM SPSS Amos 24 User’s Guide. IBM Software Group; 2016.

[20] IBM Corp . IBM SPSS Statistics for Windows, Version 26.0. IBM Corp; 2019.

[21] Zaikina A, Shustov D, Fedotov I. Sexual attitudes and gender differences in traditional societies: a cross-sectional analysis. Sexual Medicine. 2021;9(1):100293.

[22] Ahlborg T, Misvaer N, Möller A. Perception of strategy and predictors of sexual satisfaction of 1,366 parents of small children in Sweden. Journal of Sexual Medicine. 2009;6(7):1888–1900.

