# Supplementary material for: Predictors of sexual attitudes in arranged marriages: the mediating role of sexual intercourse frequency
Source: Sex Med. 2026 Sep 30;14(6):qfag085. doi: 10.1093/sexmed/qfag085 (PMC13625645; doi:10.1093/sexmed/qfag085)
Supplement: Supplementary file 1 [file supplementary_material_qfag085.zip › STROBE_checklist_cross-sectional.pdf]

STROBE Statement—Checklist of items that should be included in reports of ***cross-sectional studies***

	Item No	Recommendation	Page No
**Title and abstract**	1	(*a*) Indicate the study's design with a commonly used term in the title or the abstract	p.1
		(*b*) Provide in the abstract an informative and balanced summary of what was done and what was found	p.1
**Introduction**			
Background/rationale	2	Explain the scientific background and rationale for the investigation being reported	P.2.
Objectives	3	State specific objectives, including any prespecified hypotheses	p.2<br>p 3
**Methods**			
Study design	4	Present key elements of study design early in the paper	P3
Setting	5	Describe the setting, locations, and relevant dates, including periods of recruitment, exposure, follow-up, and data collection	P3
Participants	6	(*a*) Give the eligibility criteria, and the sources and methods of selection of participants	P3
Variables	7	Clearly define all outcomes, exposures, predictors, potential confounders, and effect modifiers. Give diagnostic criteria, if applicable	P4
Data sources/ measurement	8*	For each variable of interest, give sources of data and details of methods of assessment (measurement). Describe comparability of assessment methods if there is more than one group	P5
Bias	9	Describe any efforts to address potential sources of bias	P5
Study size	10	Explain how the study size was arrived at	P5
Quantitative variables	11	Explain how quantitative variables were handled in the analyses. If applicable, describe which groupings were chosen and why	P5
Statistical methods	12	(*a*) Describe all statistical methods, including those used to control for confounding	P6
		(*b*) Describe any methods used to examine subgroups and interactions	P6
		(*c*) Explain how missing data were addressed	P6
		(*d*) If applicable, describe analytical methods taking account of sampling strategy	N\A
		(*e*) Describe any sensitivity analyses	N\A
**Results**			
Participants	13*	(a) Report numbers of individuals at each stage of study—eg numbers potentially eligible, examined for eligibility, confirmed eligible, included in the study, completing follow-up, and analysed	P6
		(b) Give reasons for non-participation at each stage	P6
		(c) Consider use of a flow diagram	P6
Descriptive data	14*	(a) Give characteristics of study participants (eg demographic, clinical, social) and information on exposures and potential confounders	P6
		(b) Indicate number of participants with missing data for each variable of interest	P6
Outcome data	15*	Report numbers of outcome events or summary measures	P7

Main results	16	(*a*) Give unadjusted estimates and, if applicable, confounder-adjusted estimates and their precision (eg, 95% confidence interval). Make clear which confounders were adjusted for and why they were included	P7
		(*b*) Report category boundaries when continuous variables were categorized	P7
		(*c*) If relevant, consider translating estimates of relative risk into absolute risk for a meaningful time period	P7
Other analyses	17	Report other analyses done—eg analyses of subgroups and interactions, and sensitivity analyses	P7
**Discussion**			
Key results	18	Summarise key results with reference to study objectives	P8
Limitations	19	Discuss limitations of the study, taking into account sources of potential bias or imprecision. Discuss both direction and magnitude of any potential bias	P8
Interpretation	20	Give a cautious overall interpretation of results considering objectives, limitations, multiplicity of analyses, results from similar studies, and other relevant evidence	P8
Generalisability	21	Discuss the generalisability (external validity) of the study results	P8
**Other information**			
Funding	22	Give the source of funding and the role of the funders for the present study and, if applicable, for the original study on which the present article is based	P9

*Give information separately for exposed and unexposed groups.

Note: An Explanation and Elaboration article discusses each checklist item and gives methodological background and published examples of transparent reporting. The STROBE checklist is best used in conjunction with this article (freely available on the Web sites of PLoS Medicine at http://www.plosmedicine.org/, Annals of Internal Medicine at http://www.annals.org/, and Epidemiology at http://www.epidem.com/). Information on the STROBE Initiative is available at www.strobe-statement.org.
