# Supplementary material for: Predictors of sexual attitudes in arranged marriages: the mediating role of sexual intercourse frequency
Source: Sex Med. 2026 Sep 30;14(6):qfag085. doi: 10.1093/sexmed/qfag085 (PMC13625645; doi:10.1093/sexmed/qfag085)
Supplement: Supplementary file 1 [file supplementary_material_qfag085.zip › Supplemantary_File_1 (1)_qfag085.docx]

# Supplementary File 1

## Study Questionnaire

### Predictors of Sexual Attitudes in Arranged Marriages in Turkey: The Mediating Role of Sexual Intercourse Frequency

## Part I. Researcher-Developed Sociodemographic and Marital Characteristics Questionnaire

Participants completed a researcher-developed questionnaire to obtain sociodemographic and marital information before completing the Hendrick Sexual Attitudes Scale.

### Sociodemographic Characteristics

**1. Age**

- 18–29 years
- 30–39 years
- ≥40 years

**2. Gender**

- Female
- Male

**3. Educational Level**

- High school or below
- University degree or higher

**4. Employment Status**

- Employed
- Unemployed

**5. Perceived Socioeconomic Status**

- Low
- Middle
- High

**6. Place of Residence**

- Urban
- Rural

### Marital Characteristics

**7. Duration of Marriage**

- <5 years
- 5–10 years
- 10 years

**8. Do you have children?**

- Yes
- No

**9. Frequency of Sexual Intercourse**

- ≥1 time per week
- 1–3 times per month
- <1 time per month

**10. Mode of Arranged Marriage**

- Family-mediated
- Friend-mediated
- Online-assisted

# Part II. Hendrick Sexual Attitudes Scale (HSAS)

### Response Format

Please indicate the extent to which you agree with each statement.

1 = Strongly Agree

2 = Agree

3 = Neither Agree nor Disagree

4 = Disagree

5 = Strongly Disagree

## A. Permissiveness

1. I do not have to be emotionally attached to someone to have sex with them.
2. Casual sex is an acceptable experience.
3. I enjoy having sexual relationships with multiple partners.
4. One-night stands can sometimes be enjoyable.
5. It is acceptable to have sexual relationships with more than one person at the same time.
6. If both partners agree, sex can simply be a sharing of pleasure.
7. The best sex is sex without rules.
8. Life would be easier if people could engage in sexual activity more freely.
9. It is possible to enjoy having sex with someone you do not like very much.
10. Sex can simply be a form of physical relaxation.

## B. Birth Control

11. Birth control is an essential part of responsible sexuality.
12. Women should share responsibility for birth control.
13. Men should share responsibility for birth control.

## C. Communion

14. Sex is the closest form of communication between two people.
15. Sexual intercourse between two people who deeply love each other represents the highest level of human intimacy.
16. When sex is of high quality, it resembles the union of two souls.
17. Sex is a very important part of life.
18. Sex is often an intense and overwhelming experience.

## D. Instrumentality

19. The best sex is when you focus on your own pleasure.
20. The primary purpose of sex is to obtain pleasure from another person.
21. The main purpose of sex is pleasure.
22. Sex is primarily a physical act.
23. Sex is fundamentally a bodily function, similar to eating.

## Scoring

The Hendrick Sexual Attitudes Scale consists of **23 items** grouped into four dimensions:

- **Permissiveness** (Items 1–10)
- **Birth Control** (Items 11–13)
- **Communion** (Items 14–18)
- **Instrumentality** (Items 19–23)

Responses are scored on a five-point Likert scale. Higher scores indicate more positive or permissive sexual attitudes.

**Response options:**
**A = Strongly Agree; B = Agree; C = Neither Agree nor Disagree; D = Disagree; E = Strongly Disagree.**

## References

Hendrick SS, Hendrick C, Reich DA. The Brief Sexual Attitudes Scale. Journal of Sex Research. 2006;43(1):76–86.

The Turkish version of the Hendrick Sexual Attitudes Scale used in this study was administered in its validated form.
